# Robust evaluation of 3D electron cryomicroscopy data using tilt-pairs

**DOI:** 10.1016/j.jsb.2014.06.006

**Published:** 2014-08

**Authors:** Christopher J. Russo, Lori A. Passmore

**Affiliations:** MRC Laboratory of Molecular Biology, Francis Crick Avenue, Cambridge CB2 0QH, UK

**Keywords:** Electron cryo-microscopy, Single-particle reconstruction, Structure validation, Cryo-EM, Protein structure

## Abstract

Determining the structure of a protein complex using electron microscopy requires the calculation of a 3D density map from 2D images of single particles. Since the individual images are taken at low electron dose to avoid radiation damage, they are noisy and difficult to align with each other. This can result in incorrect maps, making validation essential. Pairs of electron micrographs taken at known angles to each other (tilt-pairs) can be used to measure the accuracy of assigned projection orientations and verify the soundness of calculated maps. Here we establish a statistical framework for evaluating images and density maps using tilt-pairs. The directional distribution of such angular data is modelled using a Fisher distribution on the unit sphere. This provides a simple, quantitative and easily comparable metric, the concentration parameter *κ*, for evaluating the quality of datasets and density maps that is independent of the data collection and analysis methods. A large *κ* is indicative of good agreement between the particle images and the 3D density map. For structure validation, we recommend κ>10 and a *p*-value <0.01. The statistical framework herein allows one to objectively answer the question: Is a reconstructed density map correct within a particular confidence interval?

## 1 Introduction

Single particle electron microscopy (EM) can be used for three-dimensional (3D) structure determination of biological macromolecules. With the advent of direct electron detectors, more stable stages and reliable microscopes with field emission guns, near atomic resolution structures are now possible in the best cases (Kuhlbrandt, 2014). Still, important biological information can be obtained from medium resolution (10–50 Å) density maps where the secondary structure of the molecules is not resolved.

In single particle EM, two dimensional (2D) projection images of biological specimens are recorded in an electron microscope, their relative orientations are determined using one of a number of alignment algorithms, and finally one or more 3D reconstructions are calculated (Frank et al., 1996, Van Heel et al., 1996, Marabini et al., 1996, Grigorieff, 2007, Tang et al., 2007, Scheres, 2012). With favourable datasets (high signal-to-noise, even particle distributions, homogeneous conformation, etc.), iterative refinement of the orientations assigned to each particle image will converge to the true 3D density map. But because biological specimens are radiation sensitive, imaging takes place under low-dose conditions resulting in low signal-to-noise images. Moreover, complex heterogeneity, blurring of particle images due to radiation-induced motion and unfavourable protein interactions with surfaces degrade image quality. Obtaining an initial model that is suitable for accurate refinement of orientation parameters also remains a major challenge, especially for molecules lacking distinct low-resolution structural features (Henderson et al., 2011, Henderson and McMullan, 2013, Elmlund et al., 2013). Thus, in unfavourable cases, the refinement procedure can converge to a local minimum with an incorrect 3D map (Stewart and Grigorieff, 2004, Scheres and Chen, 2012, Murray et al., 2013, Henderson, 2013). It is therefore important to independently validate whether the resultant 3D density map is correct.

Analysis of pairs of particle images recorded at different tilt angles (tilt-pairs) provides an objective measure of the accuracy of particle alignment and the validity of reconstructed maps that is not subject to the problems associated with over-fitting of noisy data (Rosenthal and Henderson, 2003, Henderson et al., 2011). Tilt-pair data are easily collected with any single particle dataset, and are evaluated by determining whether the independently assigned orientation parameters from each tilt-pair match the known tilt angle and direction (Wasilewski and Rosenthal, 2014). Ideally, the calculated tilt angle and tilt direction would be located close to the true tilt angle and direction of the goniometer for all particles. Although this is true for large complexes that align well (e.g. rotavirus with molecular weight ~50 MDa), many smaller specimens show a large scatter of directions (Henderson et al., 2011). In such cases, it can be difficult to decide whether the clustering of points is adequate to validate a given 3D map.

A robust statistical analysis of the tilt-pair data could provide a rapid assessment of image and map quality that could be used to improve data collection and processing, and could be reported along with the structure, much as the free *R* parameter is used to asses the quality of crystal structures (Brunger, 1992). The discrete angular data generated by tilt-pair analysis comprise a distribution of directions on the unit sphere, thus making them well suited for analysis using the calculus of directional statistics. The statistics of directions is well established in several fields, and can provide rigorous and quantitative answers to important questions about experimental data quality and validity (Fisher et al., 1987, Mardia and Jupp, 2000, Tauxe, 2010). With this in mind, given one or more tilt-pair datasets, we provide methods to answer the following practical questions using statistical tests:

1. Is a particular set of tilt-pair measurements randomly distributed (and therefore should the corresponding dataset or map be discarded due to poor quality)?
2. Given a set (or sets) of tilt-pairs, is dataset *A* better than dataset *B*? or is map *A* better than map *B*?
3. Does a given dataset and map show evidence of systematic bias not assumed during the generation of the map or angular assignments?
4. Is a reconstructed density map correct to within a specified level of confidence?

## 2 Methods

### 2.1 Statistical model

**Fig.1:**
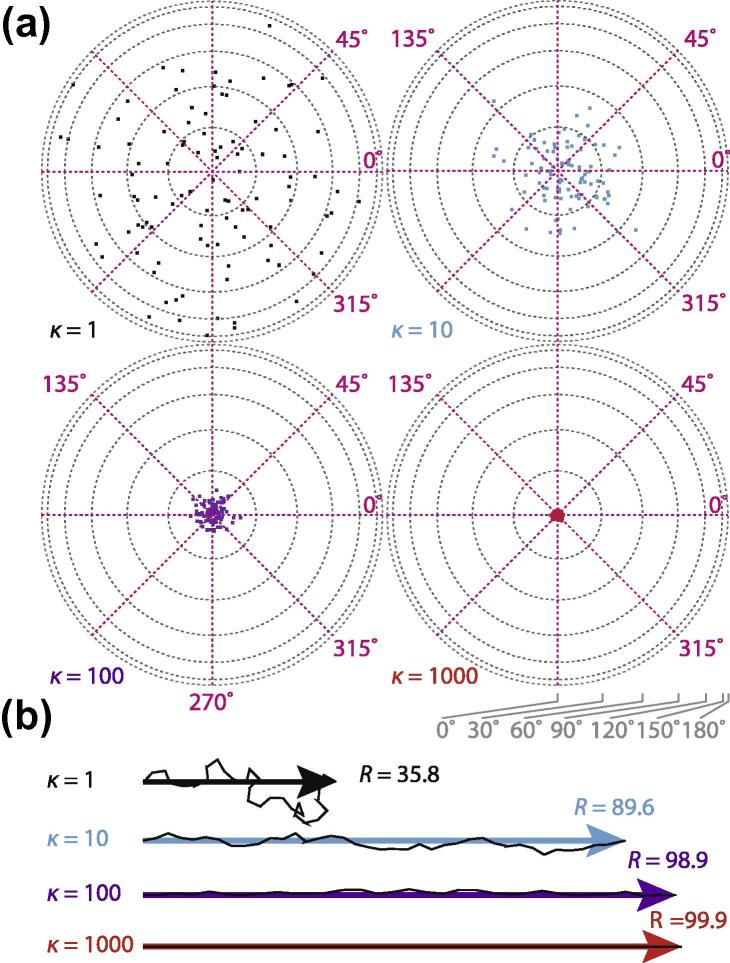
Fisher distributions using 100 simulated data points. Panel (a) shows four Fisher distributions on the unit sphere plotted using Lambert equal area projections for various concentration parameters, *κ*. For illustration, the mean direction is the pole of the sphere, which points out of the page. In the plots, the radius indicates the angle *θ* from 0° at the centre to 180° at the edge, and the azimuth indicates the direction of the tilt. Panel (b) shows a graphical construction of the *R* parameters for the same *κ* values in (a). Black segments are cartoons meant to illustrate how the individual direction vectors sum to a longer *R* as their directions become more correlated with each other. Lengths of *R* are proportional to the actual values for the distributions in (a), with the values indicated.

To analyse a particular set of tilt-pair measurements we model the distribution of directions as a Fisher distribution on the unit sphere (Fisher, 1953). The Fisher distribution is one in which the probability of an observed direction has a density

(1) $$f(\omega )\propto e^{\kappa \cos\;\omega }$$

where *ω* is the angle between the observed and the true direction. The precision parameter *κ* is the concentration of the distribution and is analogous to the inverse of the width of the Gaussian distribution. A *κ* of 0 indicates a uniform probability in all directions; as κ→∞ the distribution becomes more sharply peaked around the mean direction. Four pseudo-random samples of 100 points, taken from Fisher distributions with κ={1,10,100,1000}, are shown in Fig. 1(a).

To find the mean direction given a set of *N* tilt-pair angles $\left(\theta_{1}\text{,}\phi_{1})\ldots (\theta_{N}\text{,}\phi_{N}\right)$, where $\left(\theta_{i}\text{,}\phi_{i}\right)$ is the azimuth and inclination of a particular tilt-pair, first we convert each of the angles from spherical polar coordinates $\left(\theta_{i}\text{,}\;\phi_{i}\right)$ to vectors in Cartesian coordinates on the unit sphere:

(2) $$\left(x_{i}\text{,}y_{i}\text{,}z_{i})=(\sin\;\theta_{i}\cos\;\phi_{i}\text{,}\sin\;\theta_{i}\sin\;\phi_{i}\text{,}\cos\;\theta_{i}\right)$$

Next, we calculate the magnitude of the sum of each of the vector components over all tilt-pair angles

(3) $$R=\sqrt{{\left(\underset{i}{\sum }x_{i}\right)}^{2}+{\left(\underset{i}{\sum }y_{i}\right)}^{2}+{\left(\underset{i}{\sum }z_{i}\right)}^{2}}$$

The mean direction of the Cartesian component vectors is then

(4) $$(\overset{¯}{x}\text{,}\overset{¯}{y}\text{,}\overset{¯}{z})=\left(\frac{1}{R}\underset{i}{\sum }x_{i}\text{,}\frac{1}{R}\underset{i}{\sum }y_{i}\text{,}\frac{1}{R}\underset{i}{\sum }z_{i}\right)$$

We convert these back to an inclination and azimuth to find the mean tilt direction:

(5) $$(\overset{¯}{\theta }\text{,}\overset{¯}{\phi })=\left(\arccos\overset{¯}{z}\text{,}\arctan\frac{\overset{¯}{y}}{\overset{¯}{x}}\right)$$

The mean direction obtained from Eq. (5) represents an estimate of the true tilt direction based on the available data. Other estimates of the true direction are possible and we consider more below. The uncertainty in the mean direction as an estimate of the true direction can be represented by a confidence interval about the mean. Given that the data are taken from a Fisher distribution, we calculate the confidence interval for a given *p*-value, which is represented by a cone of solid angle around the mean direction that intersects the sphere in a circle with radius

(6) $$\alpha_{c}=\arccos\left\{1-\frac{N-R}{R}\left[{\left(\frac{1}{p}\right)}^{1/N-1}-1\right]\right\}\text{.}$$

Next we calculate the concentration (precision) parameter of the distribution, *κ*, using the approximation (Fisher, 1953)

(7) $$\kappa ≃k=\frac{N-1}{N-R}$$

which we have tested using simulations (Section 2.3) and verified for $10⩽N⩽10^{6}$ and $1⩽\kappa ⩽10^{6}$.

Finally, we calculate the median direction on the sphere (Fisher, 1985). Analogous to the linear median, the geometric median direction is defined as the location on the sphere where the sum of distances to all the points in the distribution is minimised. Various distance functions on the unit sphere can be used for this calculation; we chose the magnitude of the vector distance between the two points on the sphere. For a particular point $\left(\theta_{i}\text{,}\phi_{i}\right)$ on the sphere, the distance to the candidate median direction $\left(\theta^{\prime }\text{,}\phi^{\prime }\right)$ is

(8) $$D(\theta_{i}\text{,}\phi_{i}\text{,}\theta^{\prime }\text{,}\phi^{\prime })=\sqrt{{\left(x^{\prime }-x_{i}\right)}^{2}+{\left(y^{\prime }-y_{i}\right)}^{2}+{\left(z^{\prime }-z_{i}\right)}^{2}}\text{.}$$

Thus the median direction $\left(\overset{\sim }{\theta }\text{,}\overset{\sim }{\phi }\right)$ for a set of points $\left(\theta_{1}\text{,}\phi_{1})\ldots (\theta_{N}\text{,}\phi_{N}\right)$, is the direction which minimises the function

(9) $$E=\underset{i}{\sum }D(\theta_{i}\text{,}\phi_{i}\text{,}\theta^{\prime }\text{,}\phi^{\prime })$$

It is straightforward to minimise Eq. (9) numerically to any desired degree of accuracy using Newton’s method, and this is included in the computer programs discussed below.

Using the properties of the distribution and the calculated parameters from the data, we can now write statistical significance tests to address questions 1 to 4 from above.

#### Question 1: Is a set of tilt-pairs randomly distributed?

One measure of the randomness of a given set of tilt-pairs is the length of the vector-sum parameter *R*, as defined by Eq. (3) (Watson, 1956). Fig. 1(b) illustrates the *R* parameter. Each tilt-pair direction is a vector with unit magnitude. Summing all the component vectors together gives a composite vector *R* which increases in length as the components are better aligned with each other. A perfectly random distribution of an infinite number of tilt-pairs would have R=0 (and thus a κ=0). In the opposite extreme, if all the pairs had exactly the same direction, then *R* would be identical to *N* in length. As shown in Watson (1956), one can use this to write a simple test for randomness by comparing the lengths of *R* and *N*. Specifically, Watson showed that *R* is approximately distributed as

(10) $$R≃\sqrt{\frac{N\chi_{3}^{2}}{3}}$$

where $\chi_{3}^{2}$ is a chi-squared distribution with three degrees of freedom. Using this approximation, one can write a significance test to determine whether a dataset is randomly distributed with a particular confidence. If we take the null-hypothesis to be that the data are randomly distributed, then we can determine a length of *R* for a given number of points *N*, which we call $R_{0}$, which when exceeded entails the rejection of the null hypothesis with a specific confidence *p*. Using a confidence of p=0.01 and tabulated values of the chi-squared distribution, we can use Eq. (10) to calculate a significance length, $R_{0}$, for a given number of points:

(11) $$R_{0}≃\sqrt{3.782N}$$

So if the calculated value of $R>R_{0}$, then there is a greater than 99% chance (p<0.01) that the dataset is not randomly distributed on the sphere. If the data fail this test, than it is likely that the information content in the data or the map or both, is of too poor quality for further analysis.

#### Question 2: Is dataset *A* better than *B*?

Consider two sets of tilt-pairs collected with $N_{A}$ points in dataset *A* and $N_{B}$ points in dataset *B*, with estimated concentration parameters $k_{A}$ and $k_{B}$. Now $\kappa_{A}$ and $\kappa_{B}$ are direct measures of the quality of each dataset, and so the one with the higher *κ* is better. But if there are a small (<100) number of points in each dataset, $k_{A}$ and $k_{B}$ will only be approximations to the true values of $\kappa_{A}$ and $\kappa_{B}$ and we still wish to evaluate the statistical significance of any difference. If we take the null hypothesis that both distributions have the same concentration parameter $\left(\kappa_{A}=\kappa_{B}\right)$, then any difference must be due to sampling error for the two distributions. Using a similar approximation to that of question 1, Watson (1956) showed that the quantity 2κ(N-R) is well approximated by a chi-squared distribution with 2(N-1) degrees of freedom. So the ratio of $\kappa_{A}/\kappa_{B}$ should then vary according to

(12) $$\frac{k_{A}}{k_{B}}=\frac{\mathrm{var}[2(N_{B}-1)]}{\mathrm{var}[2(N_{A}-1)]}$$

where each are variances of $\chi^{2}$ distributions with 2(N-1) degrees of freedom. The ratio $k_{A}/k_{B}$ should thus follow the *F*-distribution (Abramowitz and Stegun, 1964) if datasets *A* and *B* have the same *κ*. We can then state that dataset *A* is better than *B* (has a higher *κ*) with confidence 1-p if the ratio $k_{A}/k_{B}$ exceeds the value of the *F*-distribution for *p*. This test is primarily useful for comparing the *κ*’s of two datasets, either with a small number of points where the difference in sampling the distribution may be the limiting factor in the comparison.

Note that the same test can also be used to compare various maps against a single set of tilt-pairs, to compare one data collection method vs. another, and even to monitor the progress of a reconstruction. For initial models in particular, determining which among a set of low resolution starting maps is best to use for further refinement is a difficult problem. Comparing the *κ* of each candidate model provides an independent and objective way of choosing which model best matches the data. We discuss the applications of comparisons based on *κ* further below.

#### Question 3: Is there evidence of systematic bias?

**Fig.2:**
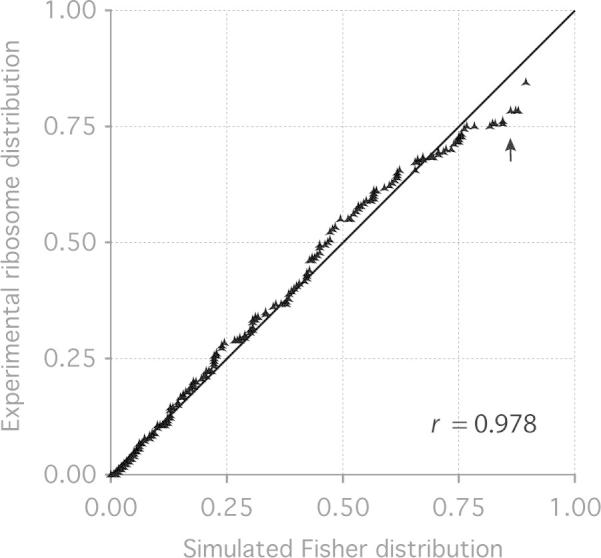
Probability plot to evaluate how well a set of tilt-pairs follows a Fisher distribution. The distribution with respect to tilt angle (*θ*) of 15 202 tilt-pairs of 70S ribosomes is plotted vs. a simulated Fisher distribution with the same *κ* and mean direction. The Pearson correlation coefficient, *r*, between the real and simulated data is 0.978. The arrow indicates the presence of a small population of outliers in the tail of the distribution (outer quantiles) as discussed in the text.

In this case, we take the null hypothesis to be that the particles examined relative to the reference map in the tilt-pairs do not follow a Fisher distribution with one mean direction. We can look for violation of the null hypothesis and thus evaluate how well the actual tilt-pair data follow a Fisher distribution. A probability plot then compares the real data against values expected for a Fisher distribution with the same *κ* and $\left(\overset{¯}{\theta }\text{,}\;\overset{¯}{\phi }\right)$ (Fisher et al., 1987). An example of this is shown in Fig. 2 for a large set of 70S ribosome tilt-pairs from Bai et al. (2013). To construct the plot, we bin the tilt-pairs using their tilt-angle, *θ*, into N+1 bins, sort them with respect to the number of points in each bin and then plot them versus a simulated Fisher distribution with the same parameters and binned in the same way. For data that are a perfect match to a Fisher distribution, the points will all fall on a straight line with slope one, thus violating the null hypothesis. The degree to which the real data vary from this, which is easily calculated using regression analysis, yields the appropriate correlation coefficient between the real data and an ideal Fisher distribution. Evaluation of plots such as this are also useful to look for some form of non-Fisher distributed systematic error in the data. For the data in Fig. 2, we show the probability plot (with respect to *θ*) showing a correlation of 0.978 between the ribosome data and the simulated Fisher distribution. This demonstrates that the real data is well modelled by the distribution. Note that the tail of the distribution shows some deviation from the simulated data (arrow). We believe these points represent outliers and we discuss their origin and interpretation below. In the computer programs described below, we include robust estimation methods which are less sensitive to the presence of outliers to improve the accuracy of the calculation of *κ* and representative direction, which are effective even for small datasets with a significant portion of outliers.

#### Question 4: Given a set of tilt-pairs, is a map valid?

Given a reconstructed density and an independently collected set of tilt-pair images, we wish to calculate whether the map is correct to within a given confidence interval. If the known direction of tilt is $\left(\theta_{T}\text{,}\;\phi_{T}\right)$ and has negligible error relative to the angular accuracy, then if $\left(\theta_{T}\text{,}\;\phi_{T}\right)$ is within the circle $\alpha_{c}$ for a given *p*-value (Eq. (6)), and the circle does not also include the untilted direction (typically assigned to (0, 0) during calculation) then the map is correct with confidence >1-p. The *p*-value assigns a statistical significance to the difference between two angles ((0, 0) and $\left(\theta_{T}\text{,}\;\phi_{T}\right)$) and so must exclude the untitled direction for the tilt-pair test.

Using this significance test, we can now define a “passed tilt-pair test” as a known tilt angle, $\left(\theta_{T}\text{,}\;\phi_{T}\right)$ falling within the circle defined by $\alpha_{c}$ around the mean direction of the independently measured tilt-pair distribution $\left(\overset{¯}{\theta }\text{,}\;\overset{¯}{\phi }\right)$ which simultaneously excludes the untilted direction. While a confidence interval of 99% is likely sufficient to remove reasonable doubt from most maps, the specific confidence interval, *p*-value, the number of tilt-pairs, *N*, and the precision parameter, *κ*, should always be included with any claims of a passed test.

### 2.2 Computer programs

While the mathematical framework here is straightforward to implement in any high-level programming language, we have written simple and efficient computer programs in ANSI-C which calculate the parameters of the Fisher distribution which best fit datasets of tilt-pair angles. The source code is freely available via a website maintained by the authors: http://www.mrc-lmb.cam.ac.uk/tiltstats/. The primary program, called **tiltStats** calculates the κ,θ and *ϕ* parameters for a given set of tilt-pairs, and includes mean direction, geometric median and can handle the presence of a significant number of outliers using Winsorized versions (Tukey, 1962) of all the estimators. The program for simulating pseudo-random datasets taken from a Fisher distribution with a given set of input parameters, which was used to generate the distributions in Fig. 1 and perform the Monte-Carlo simulations below, is called **simFisher**. A program for creating equal area projection maps is included as **projectEqA**. Equal area projections are the preferred method of plotting tilt-pair direction data–in contrast to standard polar plots–as they do not cause distortions that can make the density of points appear more concentrated than they actually are. A utility for performing coordinate transformations on the unit sphere called **rotateData**, which is useful for rotating the untilted direction to the pole, for example, is also included. Output data is available in plain text and STAR formats (Hall and McMahon, 2010). Finally, scripts for converting the tilt-pair output from Tiltdiffmulti (Rosenthal and Henderson, 2003, Henderson et al., 2011), EMAN2 (Tang et al., 2007), Xmipp (Marabini et al., 1996) and Relion (Scheres, 2012) output files for direct input into **tiltStats** are included.

### 2.3 Error in the calculation of parameters

To demonstrate the amount of error one can expect in the precision parameter for a particular number of tilt-pairs, we performed Monte-Carlo type simulations of pseudo-random data taken from Fisher distributions with a range of different *κ* values. Each simulation was run 1000 times. We then used **tiltStats** to calculate the *k* for each set and found the standard deviation in the values obtained. As expected, the error in the measurement of *κ* generally scales with a Poisson-like statistic as it is inversely proportional to $\sqrt{N}$ but is not dependent on the value of *κ*. The standard deviation of *κ* is ∼10% for 100 tilt pairs, ∼3% for 1000 tilt-pairs, and ∼1% for 10 000 tilt-pairs. This analysis is therefore a useful guide to the appropriate number of tilt-pairs required to achieve a particular level of accuracy in the measurement of *κ*. For well aligned complexes like ribosome or rotavirus, the error in *κ* for a tilt-pair dataset of 100 angles should be of order 10%. We further note that while the radius $\alpha_{c}$ for a particular p-value depends on the number of points *N* (Eq. (6)), the value of *κ* does not as it is a property of the underlying distribution, not how well we have measured it. This entails that lower quality datasets (low *κ*) will be harder to validate at a given confidence interval, and more points or larger tilt angles will be required.

## 3 Results and discussion

### 3.1 Analysis of cryo-EM datasets

**Fig.3:**
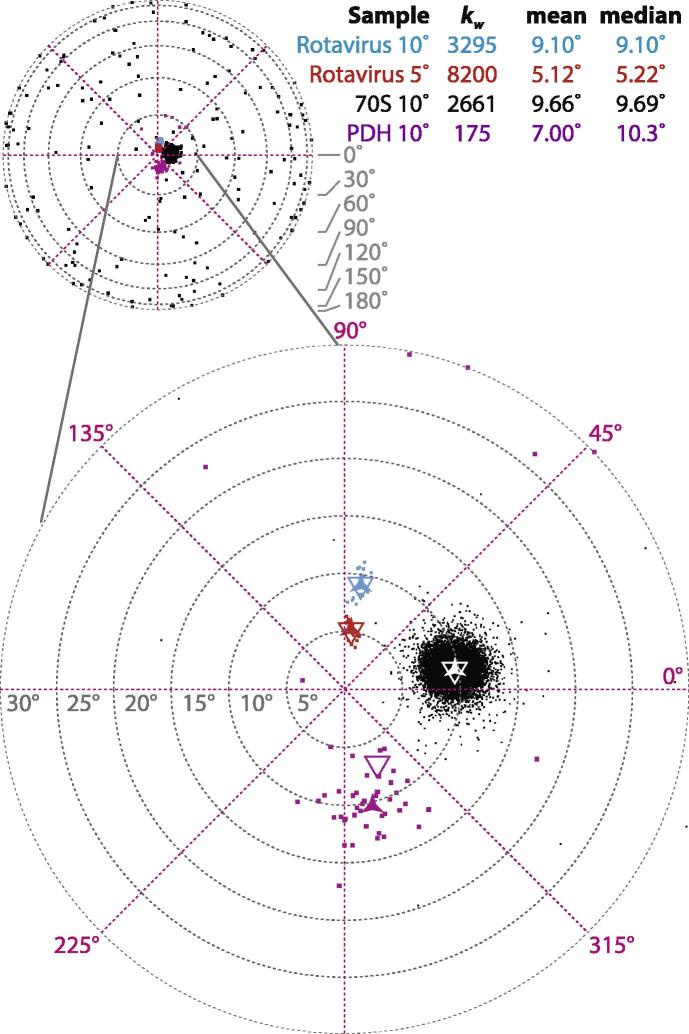
Equal area projection plots and Fisher parameters of various cryo-EM datasets. Each point is the direction on the unit sphere and the mean (triangle) and median (star) directions for each distribution are shown. The Winsorized estimate of $\kappa \text{,}\;k_{w}$, for each distribution as well as the mean and median are tabulated.

To demonstrate the application of these statistical analysis methods on real tilt-pair datasets, we selected four previously published datasets which were expected to have a range of different *κ* values and which contained varying numbers of datapoints (Henderson et al., 2011, Bai et al., 2013). The results are shown in Fig. 3. Each solid dot is one tilt-pair, the triangle is the mean direction and the solid star is the median direction. The two rotavirus datasets, one tilted to 5° (red) and one to 10° (blue), show the highest precision parameters (*κ*) of 8200 and 3295 respectively. This is not surprising as rotavirus is a large symmetric particle that can be aligned with sub-degree accuracy. In addition, the mean and median directions are almost identical, as there appear to be no outliers in either dataset. The data at 10° is the composite of two micrographs, and differing directions of radiation-induced motion for the two micrographs likely account for increased spread. In contrast, the rotavirus data at 5° were from a single micrograph where the complex rotated little, thus resulting in the high degree of precision and the high value of *κ*.

The 70S ribosome data set shows a slightly lower value for κ=2661 and comprises the data used in Fig. 2. This is concordant with the fact that the ribosome (molecular weight 2.6 MDa), while not as large as the virus particles, still can be aligned with a high degree of accuracy. Since the ribosome dataset is large (15k particles), the presence of some outliers (black dots all over the sphere visible in the unmagnified view) has little detrimental effect on the measurement of the mean direction, which agrees well with the median. We note that the accuracy of *k* for these data was improved by reducing the effect of the outliers by Winsorizing (Tukey, 1962) the data ($k_{w}$), which we discuss below. Finally, the pyruvate dehydrogenase (PDH) dataset (purple) was the first tilt-pair data published (Rosenthal and Henderson, 2003), and has a $k_{w}$ of 175. This reflects the increased difficulty in aligning these smaller particles (molecular weight 1.6 MDa) relative to the other specimen. We also note the significant difference between the mean and median directions in this dataset. Several of the points in these data likely represent outliers from the distribution. Since more of the outliers happen to fall on one side of the true direction, the mean direction is pulled further away from its true value. Separation between the mean and median values of a distribution often points to the presence of outliers, and in this case the geometric median direction is generally the better estimator of the true direction. We will consider the problem of outliers in more detail below.

### 3.2 Robustness to outliers

Outliers are a general problem in the statistical analysis of real data, and there are a multitude of “robust” mathematical methods to reduce their detrimental effect on the measurement of parameters. Here we consider the most likely origin of the outliers in tilt-pair data and provide appropriate robust methods for estimating Fisher distribution parameters in data that contain outliers.

For any individual protein particle taken from a large sample population, it is possible that the particle is partly denatured, missing a subunit, in close proximity to contamination, or somehow significantly damaged relative to the ensemble average particle used to generate the reference map. Any of these may cause at least one of the pair of images to be incorrectly aligned. These or other unmodeled measurement errors may account for the outliers seen in experimental tilt-pair distributions. This occurs because once one Euler angle is incorrectly assigned to one image of the pair, the difference in the two angles has a uniform probability of being found anywhere on the sphere, thus violating the Fisher model for directions.

Removing outliers from the distribution improves the accuracy of parameter estimation, particularly for the value of *κ*, but must be done with care to avoid incorrectly removing real data points. Rosenthal and Henderson (2003), recommend that those particles whose tilt-angles are in a plane that is not consistent with the known plane of the camera relative to the goniometer are discarded as outliers. We agree with this strategy as these points will not be correctly modelled during analysis. Still, as the Euler angle difference of an outlier can essentially be anywhere, this will not rigorously remove all the potential outliers from a distribution. A commonly used method for reducing the effect of outliers without removing data is Winsorization (Fisher, 1982), where the outer quantiles in a distribution beyond some cutoff are assigned to the last unmodified quantile. This avoids removing data entirely but still prevents the outliers from inappropriately skewing the parameter estimation. The cutoff for Winsorization is always somewhat arbitrary but is best determined by first calculating a probability plot like the one in Fig. 2, and then setting the threshold based on where the data begin to deviate significantly from the model. For carefully picked particles where the out-of-plane pairs have been discarded, we have found that the remaining outlier content comprises less than 10% of a typical dataset. The robust median estimator used here remains robust to as much as 50% outliers (Fletcher et al., 2009). We compare the use of robust estimators on real data below.

### 3.3 Comparing the quality of different datasets

**Fig.4:**
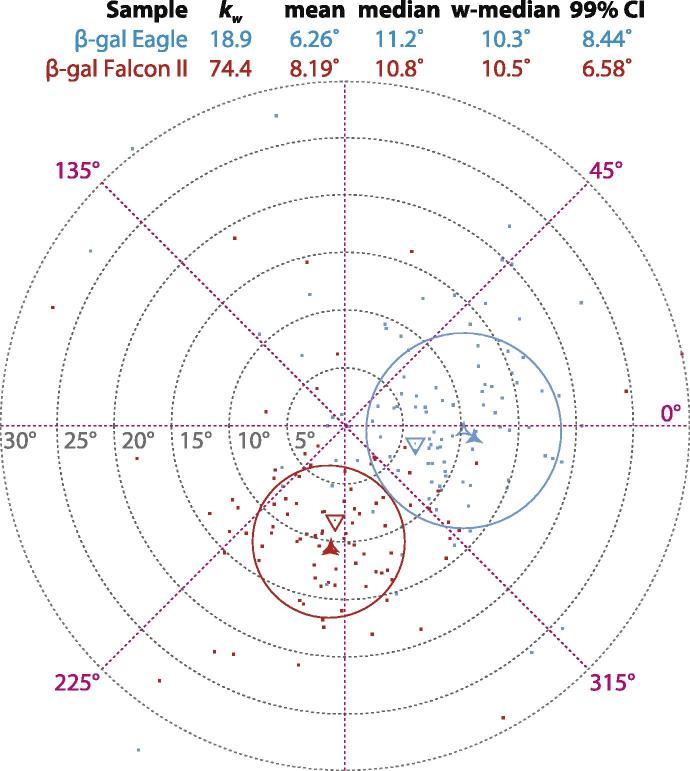
Comparison of tilt-pairs on the same *β*-galactosidase specimen imaged with two different detectors. Blue data were collected using a conventional phosphor imager, fiber-coupled to a CCD (Eagle) and red data were collected on a back-thinned direct electron detector (Falcon II). The mean (triangle), median (hollow three-pointed star) and Winsorized median (filled star) for each are shown and the 99% confidence intervals are drawn as circles of radius $\alpha_{c}$ about the median (*p* = 0.01).

To show the utility of tilt-pair statistical analysis for comparing the quality of various datasets, we calculated the Fisher parameters for two previously published datasets collected on the protein *β*-galactosidase (*β*-gal) (Henderson et al., 2011, Henderson and McMullan, 2013). The two datasets were collected using different imaging detectors: the first was a traditional phosphor imager, fibre coupled to a charge-coupled device (CCD) camera (FEI Eagle) and the second was a back-thinned direct electron detector (FEI Falcon II). The rest of the experimental parameters were otherwise the same. The respective angular distributions are plotted in Fig. 4. While the actual direction of the tilt, and even the relative quality of the two distributions is not immediately obvious just by looking at the scatter plot, the calculated Fisher parameters show that the precision of the data collected on the direct electron detector is significantly better. In particular, by using Eq. (12) we can calculate that the dataset collected on the direct detector is better than that on the phosphor (higher *κ*, and therefore more precise angular assignments) with confidence $p<10^{-10}$. This agrees with the fact that the detective quantum efficiency of the direct electron detector is significantly higher, thus leading to improved image quality and higher accuracy in particle angular determination (Henderson and McMullan, 2013). In addition, the robust estimators (median and Winsorized median) more accurately determine the correct tilt angle of 10.0° than the mean alone. We note that even though these tilt-pairs show a higher degree of scatter than those from the larger particles (viruses and ribosomes), both datasets would “pass” the tilt-pair test with a p<0.01 as defined here.

### 3.4 Angular accuracy and B-factors

Finally we wish to relate the concentration parameter *κ* to more physically relevant parameters used in cryo-EM reconstructions: angular accuracy Δθ, and *B*-factor (Rosenthal and Henderson, 2003, Henderson et al., 2011). Imperfections in particle images are often characterised using an empirical model of short-range atomic motion. This uses a Gaussian function to model the loss of high resolution spatial information from the image, where an empirically determined parameter (Debye–Waller thermal parameter or *B*-factor) provides a measure of the image quality. A number of variables can contribute to the total *B*-factor including radiation-induced particle movement, specimen charging, radiation damage and other factors that cause a loss of contrast. Errors in the assignment of angles of individual particles, Δθ, are a direct measure of the rotational blurring of the particle in the image. We define this component of the total *B*-factor as $B_{\mathrm{ang}}(B_{\mathrm{computation}}$ in Henderson et al., 2011) and calculate it below.

If we rotate the coordinate system of a Fisher distribution such that the mean direction is on the pole, then the distribution simplifies to

(13) $$f(\theta \text{;}\kappa )=\frac{\kappa }{2\sinh\;\kappa }e^{\kappa \cos\;\theta }\sin\;\theta \text{.}$$

For large values of *κ*, i.e. greater than 10, this distribution is well approximated by an exponential distribution (Fisher, 1985):

(14) $$f(\theta \text{;}\kappa )≃g(x\text{;}\kappa )=\kappa e^{-\kappa x}$$

where x=1-cosθ. We can now use the properties of the exponential distribution to find an analytic expression for the angular accuracy, Δθ, and *B*-factor due to angular error, $B_{\mathrm{ang}}$, discussed in Rosenthal and Henderson (2003). If we take the expected value of the exponential distribution as representative of the angular error, then

(15) $$\Delta \theta =\frac{1}{\sqrt{2}}\arccos\left(1-\frac{1}{\kappa }\right)$$

where Δθ is the error in each individual tilt-pair image. Note that the factor of $\sqrt{2}$ arises from the fact that the tilt-pair measurement represents the combined error of two individual angle measurements. This assumes that the error is the same for both the first and second images; in practice this will depend on many different parameters including the dose in each image, the radiation-induced motion of the particle, and the progressive radiation damage, but for the purposes of this error estimate this approximation is reasonable. Using the formula for *B*-factor due to angular accuracy derived in Rosenthal and Henderson (2003) and Eq. (15), we can then write

(16) $$B_{\mathrm{ang}}=\frac{3\pi^{2}}{40}D^{2}\arccos^{2}\left(1-\frac{1}{\kappa }\right)$$

where *D* is the diameter of the particle and the arccos is in radians. Using these formulae, we tabulate the precision, angular accuracy and *B*-factors for the data presented above, in Table 1, thus linking the precision parameter *κ* to the physical parameters important for reconstruction.

**Table 1:** Precision, angular accuracy & *B*-factors for cryo-EM data.

Complex	Mass (MDa)	*D* (Å)	*N*	*κ*	Δθ(°)	$B_{\mathrm{ang}}$ (Å^2^)
Rotavirus 5°	50	700	18	8200	0.63	88
Rotavirus 10°	50	700	14	3295	1.0	220
70S	2.6	265	15 202	2661	1.1	39
PDH	1.6	280	50	175	4.3	660
*β*-gal Falcon	0.45	135	93	74	6.7	370
*β*-gal Eagle	0.45	135	119	19	13	1400

Tilt-pair data can therefore be used not only for map validation, but also to measure physical properties of the specimen under study and to optimise methods of data collection and analysis. In particular, using this statistical framework, tilt-pairs are a quick method of comparing the quality of samples or methods that does not require calculating an entire reconstruction with a large dataset. Currently, the primary way to assess the quality of samples or methods involves completing the entire reconstruction process and comparing the final maps. While this may be the ultimate goal of an improved method, it can take many tens of hours of microscope time and years of CPU time to complete and still give ambiguous comparisons due to the often complicated process of selecting and discarding images during data processing. Using tilt-pair data collected on a specimen with known structure requires only minutes of both microscope and CPU time and provides an objective comparative metric, *κ*, of the quality of the data for comparison. Furthermore, by including a set of tilt-pairs with the rest of the particles in a particular reconstruction, one can directly monitor the progress of the reconstruction using the angular accuracy from Eq. (15). This promises to be useful as it is a truly independent measurement of the accuracy of the reconstruction in each iteration of map refinement.

## 4 Conclusions

Given the evidence presented above, we expect that tilt-pair datasets, in conjunction with robust statistical methods of modelling their angular distributions, will enable: (1) quantitative analysis, comparisons and rapid evaluation of datasets, (2) objective and comparative evaluation of sets of initial models, (3) direct quantification of the progress of reconstruction using an independent measure of angular accuracy, (4) quantitative comparisons of different microscopy techniques and methods, and (5) map validation to a particular confidence interval.

For map validation in particular, we recommend reporting the following statistical parameters, as well as an equal area plot of the direction data: *N*, the number of tilt-pairs collected; *κ*, the precision parameter of the distribution; the *p*-value of a cone of confidence around the representative tilt direction which excludes the untitled direction; the number of points which are within the cone; and the details of any outlier removal procedures such as discarding out-of-plane points or Winsorization. Using these methods, it is now possible to assign a confidence interval to a low to medium resolution 3D cryo-EM density map and thus avoid the dangers associated with over-interpreting inherently noisy data.
